# Physiological and Metagenomic Characterizations of the Synergistic Relationships between Ammonia- and Nitrite-Oxidizing Bacteria in Freshwater Nitrification

**DOI:** 10.3389/fmicb.2018.00280

**Published:** 2018-02-27

**Authors:** Mingwei Cai, Siu-Kin Ng, Chee Kent Lim, Hongyuan Lu, Yangyang Jia, Patrick K. H. Lee

**Affiliations:** School of Energy and Environment, City University of Hong Kong, Kowloon, Hong Kong

**Keywords:** freshwater, biofilter, ammonia-oxidizing bacteria, nitrite-oxidizing bacteria, synergistic relationships, metagenomics

## Abstract

Nitrification plays a crucial role in global nitrogen cycling and treatment processes. However, the relationships between the nitrifier guilds of ammonia-oxidizing bacteria (AOB) and nitrite-oxidizing bacteria (NOB) are still poorly understood, especially in freshwater habitats. This study examined the physiological interactions between the AOB and NOB present in a freshwater aquarium biofilter by culturing them, either together or separately, in a synthetic medium. Metagenomic and 16S rRNA gene sequencing revealed the presence and the draft genomes of *Nitrosomonas*-like AOB as well as *Nitrobacter*-like NOB in the cultures, including the first draft genome of *Nitrobacter vulgaris*. The nitrifiers exhibited different growth rates with different ammonium (NH_4_^+^) or nitrite concentrations (50–1,500 μM) and the growth rates were elevated under a high bicarbonate (HCO_3_^-^) concentration. The half-saturation constant (*K*_s_ for NH_4_^+^), the maximum growth rate (μ_max_), and the lag duration indicated a strong dependence on the synergistic relationships between the two guilds. Overall, the ecophysiological and metagenomic results in this study provided insights into the phylogeny of the key nitrifying players in a freshwater biofilter and showed that interactions between the two nitrifying guilds in a microbial community enhanced nitrification.

## Introduction

Nitrification, the biological oxidation of ammonia (NH_3_) to nitrate (NO_3_^-^) via nitrite (NO_2_^-^), is a vital oxidative process that links reduced and oxidized inorganic nitrogen to sustain the global nitrogen cycle in soils (Wang et al., 2015) and aquatic systems (Hou et al., 2013). This process is mainly carried out by two different but interdependent guilds of lithoautotrophic microorganisms, namely the ammonia-oxidizing bacteria (AOB) (e.g., *Nitrosomonas* and *Nitrosospira*) and the ammonia-oxidizing archaea (AOA) as one guild that convert NH_3_ to NO_2_^-^ using the key enzymes ammonia monooxygenase (encoded by the *amoCAB* genes) and hydroxylamine dehydrogenase (encoded by the *haoAB* genes) (Sedlacek et al., 2016), and the nitrite-oxidizing bacteria (NOB) (e.g., *Nitrospira* and *Nitrobacter*) as another that convert NO_2_^-^ to NO_3_^-^ with the enzyme nitrite oxidoreductase (encoded by the *nxrAB* genes) (Dionisi et al., 2002). Recent reports have also identified single organisms that are capable of performing the comammox reaction, which leads to complete nitrification from NH_3_ to NO_3_^-^ (Daims et al., 2015; van Kessel et al., 2015).

The coexistence of AOB and NOB has been demonstrated in a variety of environments (Graham et al., 2007; Keuter et al., 2011; Wang et al., 2015) and microscopic examinations of samples from nitrifying reactors have often showed AOB and NOB in physical contact with each other (Okabe et al., 1999). Previous studies have shown that nitrification rates are closely related to environmental parameters such as temperature, NH_3_ and/or bicarbonate (HCO_3_^-^) concentrations (French et al., 2012; Jiang et al., 2015). Even though AOB and NOB can function independently (Koops et al., 1991; Lücker et al., 2010), their synergistic relationships (Schink, 2002) benefit both species; for example, the growth of AOB is elevated in the presence of NOB, as the latter helps prevent the accumulation of NO_2_^-^ that could inhibit AOB (Kim et al., 2006), while oxidation of NH_3_ by AOB benefits NOB by providing a consistent energy supply as NO_2_^-^ and by maintaining NH_3_ concentrations below the toxicity threshold (Laanbroek and Gerards, 1993). In some cases, AOB benefit more than NOB (Perez et al., 2015). However, the tight coupling between AOB and NOB means that even minor perturbations in the abundance of ammonia oxidizers can lead to large changes in the abundance of nitrite oxidizers (Knapp and Graham, 2007), and subsequently to erratic nitrification activities (Graham et al., 2007).

Our current understanding of the synergistic relationships between nitrifiers arises mainly from studies of artificially constructed co-cultures of isolates (e.g., pairing *Nitrosomonas europaea* and *Nitrobacter winogradskyi*) (Laanbroek and Gerards, 1993; Perez et al., 2015). These conditions may not be representative of some of the real-life environmental conditions in a microbial community in an ecosystem. We explored the relationships between ammonia-oxidizing and nitrite-oxidizing populations found in freshwater systems by instead culturing one or both of these nitrifying guilds from a microbial community inhabiting a freshwater biofilter in a synthetic medium. The community composition was determined by 16S rRNA gene and metagenomic sequencing, with the recovery of draft genomes of the nitrifiers, including the first draft genome of *Nitrobacter vulgaris*. The ecophysiological characteristics of the cultures were studied in greater depth by testing different concentrations of NH_4_^+^ and NO_2_^-^ (50–1,500 μM), and HCO_3_^-^ (1,000 and 3,000 μM). New insights were obtained into the interactions and physiology of the freshwater AOB and NOB and clues (e.g., kinetic parameters) were provided for future manipulation of microbial interactions to enhance the efficiency of microbial nitrification processes.

## Materials and Methods

### Cultivation of Nitrifiers

The inoculum used to establish the nitrifying cultures was obtained from the biofilter of a household-size freshwater fish tank. The tank housed five small tropical fish and nitrification occurred (i.e., NO_3_^-^ was detected). The biofilter was selected for use as inoculum since the microbial community had adapted to a relatively low NH_4_^+^ condition (∼500 μM was typically measured in the influent to the biofilter) and the biofilter had been in stable operation for more than two years. The synthetic sponge filter (36 cm^2^) was inoculated (March 2014) into 60-ml serum bottles sealed with butyl rubber stoppers with 30 ml of synthetic medium containing 4 μM KH_2_PO_4_, 10 ml/L mineral salts, 0.1 ml/L selenite/tungstate solution, and 1 ml/L trace metals (Biebl and Pfennig, 1978). After autoclaving, 3 μl of filter-sterilized vitamin solution (Balch et al., 1979) were added, together with NH_4_Cl and NaHCO_3_ at final concentrations of 500 and 1,000 μM, respectively. The NOB were eliminated by amending a separate set of cultures with sodium chlorate (10 mM) (Belser and Mays, 1980) until no NO_3_^-^ was detected in subsequent transfers. The cultures with NH_4_^+^ without chlorate are referred to as ‘Culture01,’ the ones with NH_4_^+^ and chlorate are ‘Culture02,’ while the Culture01 fed with NO_2_^-^ instead of NH_4_^+^ for experimental purposes are ‘Culture03.’ Cultures were grown at 25°C (akin to the temperature of the aquarium) in the dark without shaking (Martens-Habbena et al., 2009). Late exponential-phase cultures (∼10% v/v) were routinely transferred to fresh media after an incubation period of about 5 to 7 days for 15 months (until the cultures became stable) before characterizations of the cultures were performed.

### Characterizations of the Cultures

All experiments were conducted in triplicate in 30 ml medium as described in Section “Cultivation of Nitrifiers” with 3 ml of late exponential-phase culture as inoculum. A range of NH_4_^+^ concentrations (50, 200, 500, 1,000, and 1,500 μM) was used to test the growth rates of the nitrifiers in Culture01 and Culture02. The growth of NOB was tested using a range of NO_2_^-^ concentrations (50, 200, 500, 1,000, and 1,500 μM) in Culture03. Different HCO_3_^-^ concentrations (1,000 and 3,000 μM) were tested for all cultures. The potential inhibition effects of NO_2_^-^ on the AOB in Culture02 when grown with 500 μM of NH_4_^+^ were studied by adding a range of NO_2_^-^ concentrations (500, 1,500, or 10,000 μM). Liquid samples were withdrawn at regular intervals during the exponential phase (5–24 h) to determine the NH_4_^+^, NO_2_^-^ and/or NO_3_^-^ concentrations, and the collected samples were filtered (0.2 μm) prior to storage at -80°C. The ultimate dissolved oxygen concentration and pH of the cultures were measured using a portable oximeter (SevenGo Duo Pro-SG68, Mettler Toledo, Switzerland). The dissolved oxygen concentration was more than 5.6 mg/L and pH ranged from 6.8 to 7.0 in all cultures.

### 16S rRNA Gene Amplicon Sequencing and Analysis

The composition of the microbial communities was determined by collecting a section of the biofilter (36 cm^2^) and 20 ml of Culture01 and Culture02 for DNA extraction using the PowerSoil DNA Isolation Kit (Mo Bio Laboratories, Carlsbad, CA, United States), as described previously (Lu et al., 2013). The 515F/806R universal primer pair (Caporaso et al., 2011) was used to amplify the V4 region of the 16S rRNA gene of the genomic DNA. The PCR conditions, amplicon purification, and library preparation were as described previously (Leung et al., 2014). The samples were sequenced on an Illumina MiSeq platform (Genentech Corporation, Taipei, Taiwan), which generated paired-end 250-bp reads with ∼60,000 paired-end raw reads per sample.

Reads obtained from the sequencing platform were first processed by removing the barcodes and primers, followed by alignment of the reads using FLASH (V1.2.7) (Magoč and Salzberg, 2011). The forward and reverse reads gave similar results, so the forward reads were used for analysis. The aligned sequences were filtered using the QIIME pipeline (v.1.8.0) (Caporaso et al., 2010b) with the script “split_library_fastq.py.” Chimera sequences were identified and removed with UCHIME (Edgar et al., 2011) against the GOLD database (Bernal et al., 2001). OTU formation was performed following the UPARSE pipeline (Edgar, 2013) and the dereplicated reads were clustered into OTUs at a 97% sequence similarity threshold. Singleton OTUs were removed and the remaining high-quality sequence reads were aligned with PyNAST (Caporaso et al., 2010a) against the Ribosomal Database Project (RDP, release 11.3).

### Metagenomic Sequencing and Assembly

Based on the growth profiles of the cultures, three time series samples (days 1, 3, and 5) were collected from each culture (Culture01 and Culture02) for metagenomic sequencing and to facilitate the subsequent genome binning. The samples were named according to the day in which they were collected (e.g., the sample collected on day 1 is referred to as ‘Culture01_1’ and ‘Culture02_1’). Genomic DNA was extracted as indicated in Section “16S rRNA Gene Amplicon Sequencing and Analysis” from 120 ml of each culture at each time point. DNA concentrations and quality were determined using a spectrophotometer (NanoDrop 2000; Thermo Scientific, United States).

Paired-end sequencing libraries were prepared using the Illumina HiSeq 3000/4000 PE Cluster Kit with 2 μg of DNA according to the manufacturer’s instructions. The libraries were sequenced on an Illumina HiSeq 4000 sequencer, which generated 150 bp paired-end reads at the sequencing core facility of UC Berkeley. Raw sequencing reads were trimmed and filtered with a minimum quality score of 32 using Trimmomatic (version 0.35) (Bolger et al., 2014). Read pairs with either end shorter than 80 bp were discarded. *De* *novo* assembly on the filtered read pairs was performed using the IDBA-UD assembler (Peng et al., 2012) with a maximum *k*-mer size of 100 and a minimum contig length of 1.2 kb. The six metagenomes were assembled independently. The targeted nitrifiers were more abundant in the mid-point samples (day 3) so the contigs reconstructed from the mid-point metagenome (Culure01_3 and Culture02_3) served as sequence templates for coverage estimation. Paired-end information was extracted from the SAM files generated by mapping filtered read pairs to sequence templates using bwa 0.7.1 (Li and Durbin, 2010). The taxonomic compositions of the metagenomes were determined using both the reads and assembled contigs via the MG-RAST platform as described previously (Cai et al., 2016).

### Genomes Binning of the Metagenomic Sequencing Data

We distinguished individual genomes in the metagenomes using a differential coverage binning approach similar to that reported in a previous study (Albertsen et al., 2013), with modifications to include multiple time-point samples. Coverage of the individual contig from each time point was determined by mapping the filtered reads to the respective sequence templates (Culure01_3 for Culture01 and Culture02_3 for Culture02). The contigs were binned into genome bins by plotting contig coverage estimates of any two time points or, to obtain a better resolving power, by plotting the contig coverage estimates for multiple time points with multidimensional scaling (MDS). The inclusion of three metagenomes gave the best resolution. The draft genome bins were refined based on sequence compositions (GC content and tetra-nucleotide frequencies) and taxonomic compositions. In addition, contigs not included in the previous steps or wrongly assigned were recruited to, or removed from, the genome bins according to paired-end information. Reconstructed draft genomes were compared with the results generated by the expected-maximization based method MaxBin 2.0 (Wu et al., 2016). The quality of the draft genomes (e.g., completeness and contamination) was evaluated using CheckM (Version 1.0.4) (Parks et al., 2015). Genome-wide average nucleotide identity (ANI) and average amino acid identity (AAI) analyses were calculated using the online ANI and AAI calculators (Rodriguez-R and Konstantinidis, 2016).

Protein coding genes were inferred from the assembled contigs using Prodigal (v2.60) (Hyatt et al., 2010) with metagenome mode enabled. The genes were functionally annotated by searching the gene sequence against the NCBI non-redundant database using DIAMOND (v0.8.17.79) (Buchfink et al., 2015) and submitting to the KEGG Automatic Annotation Server (Moriya et al., 2007). The hallmark gene *amoC* was partially assembled, so the region-specific Sanger sequencing primer pairs were designed for amplifying the gene. Genes related to nitrogen metabolism and carbon fixation (K numbers) were compared to those from the reference genomes *Nitrosomonas* sp. AL212 (Yuichi et al., 2011) and *N.* *winogradskyi* Nb-255 (Starkenburg et al., 2006). The comparative results were visualized using Circos (Krzywinski et al., 2009). The metabolic pathways of the nitrifier bins were manually curated and reconstructed using EC numbers as described previously (Cai et al., 2016).

### Phylogenetic Analyses

The phylogeny of the nitrifiers in the cultures was determined using both the 16S rRNA genes and nitrification-related functional genes (i.e., *amoA* and *nxrA*) by the neighbor-joining algorithm in MEGA (v 5.2) (Tamura et al., 2011). Reference 16S rRNA gene sequences of the nitrifiers were recruited by searching the keywords ‘*Nitrosomonas* 16S’ and ‘*Nitrobacter* 16S’ in the NCBI database, while the amino acid sequences of the hallmark genes were obtained by searching the keywords ‘ammonia, methane, amo, pmo or monooxygenase’ and ‘nxr, nitrite reductase or nitrite oxidoreductase.’ The recruited sequences that showed a high similarity to the genes in our samples were retained for phylogenetic calculations. For the genome tree, all complete and draft genomes of AOB and NOB were obtained from NCBI. The phylogenetic analysis was performed using PhyloPhlAn (Segata et al., 2013) with default parameters.

### Inorganic Nitrogen Measurement and Nitrification Kinetics Calculation

The concentrations of NH_4_^+^ were determined using a colorimetric assay (Bollmann et al., 2011) with a spectrophotometer (SpectraMax M2, Molecular Devices, United States), while NO_2_^-^ and NO_3_^-^ were determined using ion chromatography (Dionex-ICS-1100) with an Ion Pac AS18 4 mm × 250 mm analytical column. Growth rates of the nitrifiers were calculated based on the slope of the log-transformed concentrations of NO_2_^-^ + NO_3_^-^ for AOB or NO_3_^-^ for NOB plotted against time (h) during the exponential growth phase as described previously (Bollmann et al., 2011; French et al., 2012). The coefficients of determination (*R*^2^) of all the slopes were ≥0.99. The Monod kinetics model μ = μ_max_ (*S* / (*K*_s_ + *S*)) was used to determine the characteristics (μ_max_ and *K*_s_) of the cultures. Here, μ is the growth rate at different substrate concentration, μ_max_ is the maximum growth rate, *K*_s_ is the half-saturation constant, and *S* is the rate-limiting substrate concentration. The lag duration of a culture in an experiment was calculated from the time point immediately after inoculation to the first time point used for the growth rate calculation.

### Cell Concentration Analysis

The cell concentrations of different cultures were determined by collecting 12–15 ml of the inoculum and samples at the end of incubation and extracting the genomic DNA, as indicated above. The absence/presence of specific nitrifying populations (i.e., AOA, AOB, and NOB) was verified using PCR with the primers listed in Supplementary Table S1, according to the thermocycling protocols of the respective references. The abundance of the bacterial *amoA* gene was quantified using qPCR with the primers amoA-1F and amoA-2R (Rotthauwe et al., 1997), while the primers F1norA and R2norA (Attard et al., 2010) were used to quantify the *nxrA* gene. The qPCR was performed on a StepOnePlus Real-Time PCR Systems (Applied Biosystems) with the SYBR Green master mix, according to previous studies (Attard et al., 2010; Limpiyakorn et al., 2011). Serially diluted DNA standards were prepared for absolute gene copy quantification using purified *amoA* and *nxrA* genes PCR products according to a previous study (Ritalahti et al., 2006). Melt curves were performed with each assay to confirm the specificity of the primers. For all experimental samples, the average of biological triplicates was calculated.

### Nucleotide Sequence Accession Number

The amplicon sequences, metagenomic sequences, and the AOB and NOB draft genomes generated in this study have been deposited in the NCBI BioProject Database under accession number PRJNA343684.

## Results

### Descriptions of the Nitrifying Cultures

**FIGURE 1:**
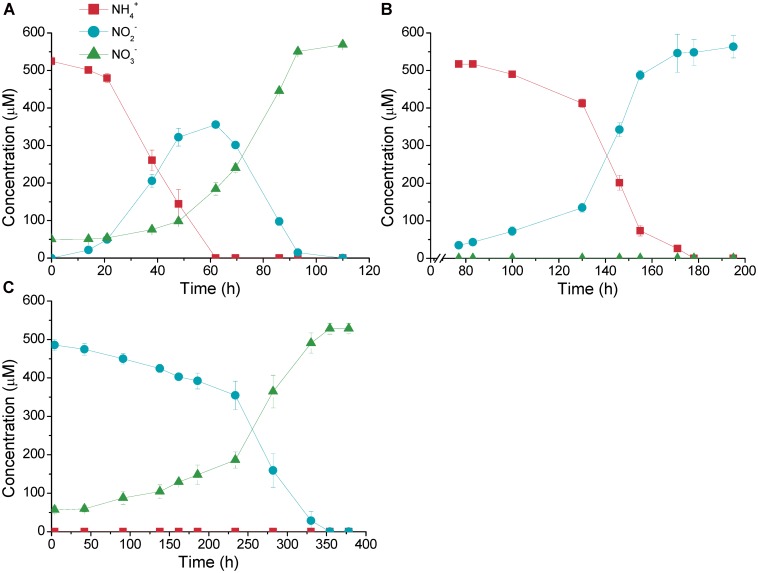
Nitrification activities of **(A)** Culture01, **(B)** Culture02, and **(C)** Culture03. The amendment was 500 μM of NH_4_^+^ (for Culture01 and Culture02) or NO_2_^-^ (for Culture03). Error bars represent one standard deviation from biological triplicate experiments.

Two cultures of nitrifiers (Culture01 and Culture02) were established from a freshwater aquarium biofilter (**Figure 1**). The concentrations of NH_4_^+^ in the influent to the biofilter were in the range of 500 μM; therefore, a synthetic medium containing 500 μM NH_4_^+^ was used to culture the nitrifiers to simulate the *in situ* process. After 15 months, a stable culture was established that converted NH_4_^+^ to NO_3_^-^ via transient production of NO_2_^-^ within 120 h (Culture01) (**Figure 1A**). Selection only for ammonia oxidizers was made by amending with chlorate, which resulted in a culture that stoichiometrically converted NH_4_^+^ to NO_2_^-^ within 180 h (including 75 h of lag time) with no NO_3_^-^ production (Culture02) (**Figure 1B**). We tested the activities of the nitrite oxidizers by transferring cells of Culture01 to a medium containing only NO_2_^-^ without NH_4_^+^, in which gave a stoichiometric conversion of NO_2_^-^ to NO_3_^-^ but required a much longer time (375 h) (Culture03) (**Figure 1C**).

### Composition of the Microbial Communities in the Biofilter and Cultures via 16S rRNA Gene Sequencing

**FIGURE 2:**
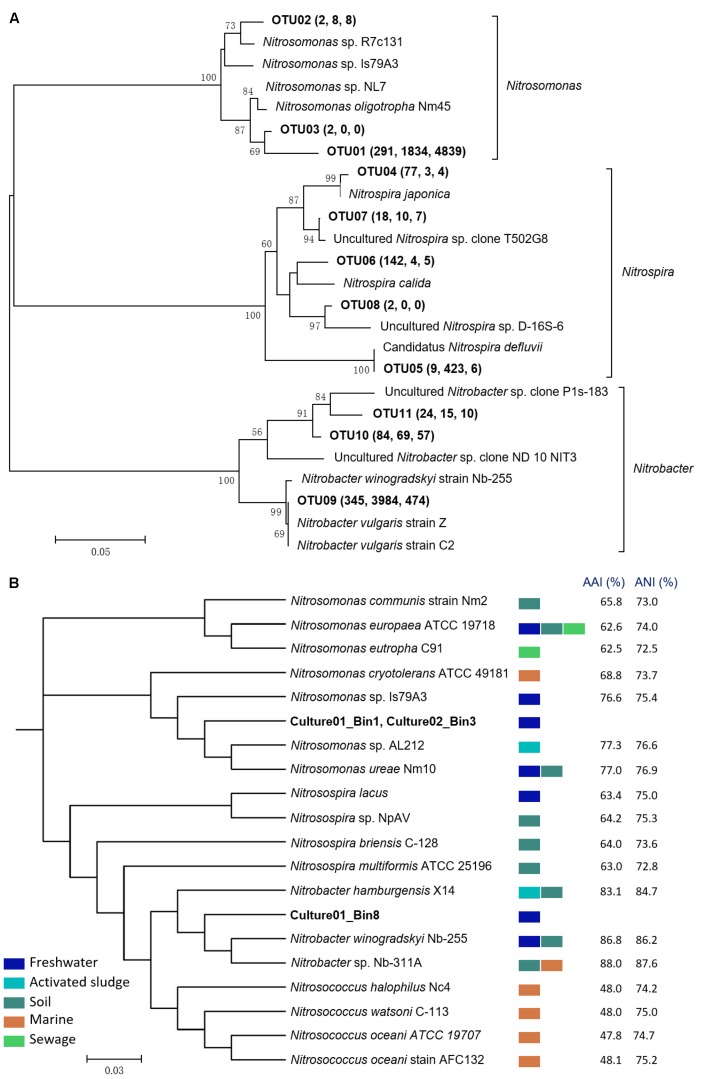
**(A)** Phylogenetic tree of the nitrifiers in the biofilter and cultures based on 16S rRNA genes. The tree was built using the neighbor-joining algorithm in MEGA 5.2. OTUs (bolded) were defined based on a similarity threshold of 97% and bootstrap values (1,000 iterations) greater than 50% are shown. The values in the brackets indicate the numbers of reads found in the biofilter, Culture01, and Culture02, respectively. **(B)** Phylogenetic tree based on the complete and draft genomes using PhyloPhlAn. AAI and ANI for *Nitrosomonas* and *Nitrosospira* were calculated against Culture01_Bin1, while *Nitrobacter* and *Nitrosococcus* were calculated against Culture01_Bin8. All internal nodes have a high bootstrap value (65–100%). Colored rectangles represent the typical habitats of the nitrifiers.

The microbial community structures of the biofilter, Culture01, and Culture02 were evaluated using 16S rRNA gene amplicon sequencing. A total of 8,728 OTUs were recovered from all the samples. After 15 months of culturing, the relative abundances of nitrifying bacteria in Culture01 and Culture02 were 19.1 and 12.4%, respectively, compared to 4.4% for the biofilter (Supplementary Figure S1). Of the OTUs pertinent to the AOB guild, three (i.e., OTU01, OTU02, and OTU03) were closely related to the *Nitrosomonas* genus. As shown in **Figure 2** and Supplementary Table S2, Culture01 and Culture02 shared the same *Nitrosomonas*-like AOB (OTU01) and this most abundant AOB clustered closely (98% sequence identity) to the 16S rRNA gene of *Nitrosomonas* *oligotropha* Nm45. The eight OTUs classified as nitrite oxidizers were distributed within the *Nitrospira* (*n* = 5; OTU04-08) and *Nitrobacter* clusters (*n* = 3; OTU09-11). Of the eight NOB-like OTUs present in the biofilter, only two increased in relative abundance in Culture01 (OTU05 and OTU09, **Figure 2A**).

An OTU of the *Nitrososphaera* family (3.6%) belonging to the AOA was found in the biofilter, but no sequence belonging to the AOA was subsequently detected in either culture. PCR with primers targeting the archaea *amoA* gene failed to detect the presence of AOA in Culture01 or Culture02 (Supplementary Table S1). Genera belonging to *Bosea* (increased by 1.5% in Culture01 and 6.1% in Culture02), *Aminobacter* (increased by 1.9% in Culture01 and 4.0% in Culture02), *Sediminibacterium* (increased by 2.5% in Culture01 and 2.6% in Culture02), and *Acidovorax* (increased by 10.0% in Culture01 and 18.4% in Culture02) were higher in both cultures relative to the biofilter (Supplementary Figure S1), while *Azospirillum* decreased (by 8.8% in Culture01 and 8.9% in Culture02). Interestingly, *Desulfitobacterium* was abundant in the biofilter (29.1%) and remained at a relatively high abundance after cultivation (20.0% in Culture01 and 16.7% in Culture02). Overall, the organisms found in the cultures were representative of the *in situ* key participants in the aquarium biofilter.

### Metagenomic Sequencing of the Cultures and Draft Genomes of the Nitrifiers

The 16S rRNA gene sequencing was supplemented with metagenomic sequencing, applied to query the genomic contents of the cultures. The variation in the community composition during growth was taken into account by collecting samples of Culture01 and Culture02 on days 1, 3, and 5, based on the nitrification profiles (**Figure 1**). The samples contained 1.0–2.8 Gb of paired-end sequences after quality control (Supplementary Table S3). The qualified reads were assembled into contigs with lengths ranging from 1.2–1,066 kb, generating a total of 45.7–59.2 Mb per sample (Supplementary Table S3). The resulting contigs were resolved and assigned into genomic bins using differential coverage binning (Supplementary Figure S2). A total of eight high-quality bins could be identified in the metagenomes of Culture01, while nine were found in Culture02 (Supplementary Table S4), with a high similarity shared between some of the bins of the two cultures (Supplementary Table S2). The high completeness (87.8–100%, except for Culture02_Bin9) and the low number of contaminating sequences found in the genomes (0–2.4%, Supplementary Table S4) suggest a high quality for the resulting bins. Furthermore, the high similarity of the bins (72.7–100%) obtained using two different binning approaches (MaxBin and differential coverage binning, Supplementary Table S5) indicates the reliability of the binning results.

Phylogenetic analysis using PhyloPhlAn based on whole genomes shows that the two AOB draft genomes (with about 97% completeness) from Culture01 (Culture01_Bin1) and Culture02 (Culture02_Bin3) are closely related to the complete genome of *Nitrosomonas ureae* Nm10 [found in freshwater and soils (Koops et al., 1991)] and *Nitrosomonas* sp. AL212 [found in activated sludge (Prosser et al., 2014)], with AAI and ANI similarities around 77% (**Figure 2B**). Pair-wise comparison of the two *Nitrosomonas*-like draft genomes in the two cultures shows an identity of more than 98% (Supplementary Table S2) and an AAI and ANI similarity of 100%. The two draft genomes of the *Nitrosomonas*-like AOB (Supplementary Table S6) contained genes encoding ammonia monooxygenase (i.e., *amoCAB* sequences). Likewise, genes encoding hydroxylamine dehydrogenase (*haoAB*) and its electron carriers, cytochromes *C*_554_ and *C*_m552,_ were identified (Supplementary Figure S3). As shown in Supplementary Figures S3, S4, the identified hallmark genes and gene loci of the *Nitrosomonas*-like AOB (*amoCAB* and *haoA*) in both cultures were highly similar to the genes of the *Nitrosomonas* cluster, consistent with the whole genome-based phylogenetic tree (**Figure 2B**), whereas they were much lower in similarity with the genes of the comammox *Nitrospira* species.

The whole genome-based phylogenetic tree also shows that a draft genome binned from Culture01 (Culture01_Bin8) was most closely related to *N.* *winogradskyi* Nb-255 [found in freshwater and soils (Koops and Pommerening Röser, 2001; Wertz et al., 2008)] and *Nitrobacter* sp. Nb-311A [found in marine and soils (Moran et al., 2007; Wertz et al., 2008)], with AAI and ANI similarities around 87% (**Figure 2B**). The draft genome of the *Nitrobacter*-like NOB (Supplementary Table S7) contained genes encoding nitrite oxidoreductase (i.e., *nxrA* and partial *nxrB* sequences) and its cognate electron carriers (i.e., cytochrome c) (Supplementary Figure S3). The sequences of the *nxrA* and *nxrB* genes and their loci were highly similar to *N.* *winogradskyi* Nb-255 and *Nitrobacter* sp. Nb-311A, in agreement with the phylogenetic tree of the whole genomes (**Figure 2B**), but very different from the comammox *Nitrospira* species (Supplementary Figure S3). Consideration of unsequenced species clustered the *nxr* genes of the cultured *Nitrobacter*-like NOB closer to *N.* *vulgaris* (Supplementary Figure S4).

Reconstruction of the key carbon metabolic pathways in the draft genomes of both the *Nitrosomonas*-like AOB (Culture01_Bin1 and Culture02_Bin3) and *Nitrobacter*-like NOB (Culture01_Bin8) revealed the presence of a complete set of genes in the metabolic pathways of both the carbon fixation and citric acid cycles, as well as acetate metabolism, which is consistent with the reference genomes from the same genus (e.g., *Nitrosomonas* sp. AL212 and *N.* *winogradskyi* Nb-255, Supplementary Figures S5, S6). The pathways for nitrogen metabolism in the draft genomes are also consistent with the reference genomes (Supplementary Figure S6).

### Physiological Activities of the Nitrifiers in the Cultures

The physiological activities of the nitrifying populations in the cultures were tested using five different concentrations (50–1,500 μM) of NH_4_^+^ or NO_2_^-^. Increasing the substrate concentrations increased the duration of the lag phase for Culture01 (23–45 h), but shortened it for Culture02 (250–120 h) (data not shown). The lag time for Culture03 increased from 88–146 h in response to increasing NO_2_^-^ concentration; this duration was longer than that observed for Culture01 with NH_4_^+^ (data not shown).

**FIGURE 3:**
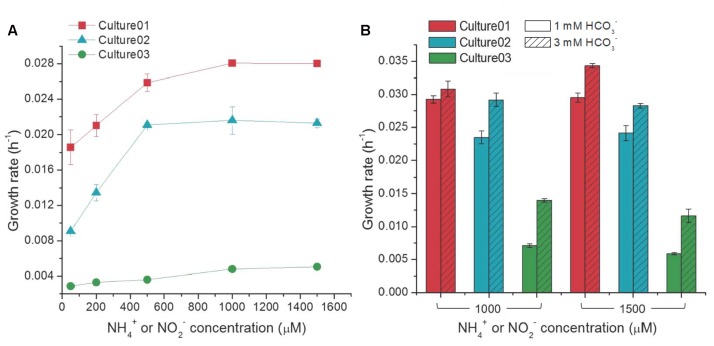
Influence of different concentrations of **(A)** NH_4_^+^ or NO_2_^-^ (with 1,000 μM HCO_3_^-^), and **(B)** HCO_3_^-^ on the growth rates of the nitrifiers. Culture01 and Culture02 were fed NH_4_^+^, while Culture03 was fed NO_2_^-^. Error bars represent one standard deviation from biological triplicate experiments.

The growth rates of Culture01 and Culture02 increased with NH_4_^+^ amendment up to 500 μM, but showed no substantial changes with higher concentrations (**Figure 3A**). Culture03 showed only incremental changes in the growth rates with increasing NO_2_^-^ concentration (**Figure 3A**). In general, the growth rates were higher for Culture01 (0.019–0.028 h^-1^) than for either Culture02 (0.0091–0.021 h^-1^) or Culture03 (0.0029–0.0051 h^-1^) (**Figure 3A**). The NO_2_^-^ inhibition test indicated that the cultured AOB in Culture02 could completely transform 500 μM NH_4_^+^ to NO_2_^-^ even following spiking with a relatively high concentration of NO_2_^-^ (10 mM). The *K*_s_ values for NH_4_^+^ uptake for Culture01 and Culture02 were 25.9 and 71.8 μM, respectively. The variations in NO_2_^-^ concentration in Culture01 during cultivation precluded an accurate determination of the activities of NOB in Culture01, while the data of Culture03 fitted the Monod kinetics model poorly.

High concentrations of NH_4_^+^ or NO_2_^-^ (1,000 or 1,500 μM) resulted in incomplete oxidation in the three cultures. The possibility of insufficient carbons and buffering capacity in the cultures was tested by amending with a higher HCO_3_^-^ concentration (3,000 μM versus 1,000 μM routinely used). The growth rates of the nitrifiers increased significantly with increasing HCO_3_^-^ concentration [Student’s *t*-test, *p* < 0.01, except Culture01 (1,000 μM NH_4_^+^)] (**Figure 3B**) and complete oxidation of the input nitrogen occurred. When compared to Culture01 and Culture02, the growth rate of Culture03 was more responsive to the increase in HCO_3_^-^ concentrations [i.e., the growth rate almost doubled in response to increasing HCO_3_^-^ concentration] (**Figure 3B**). The possible presence of carbon dioxide (CO_2_) arising from ambient air in the headspace was eliminated by purging the serum bottles with a pure gas mixture of N_2_:O_2_ (79:21%) without CO_2_ and no difference was noted with or without purging.

### Growth of Nitrifying Populations

**FIGURE 4:**
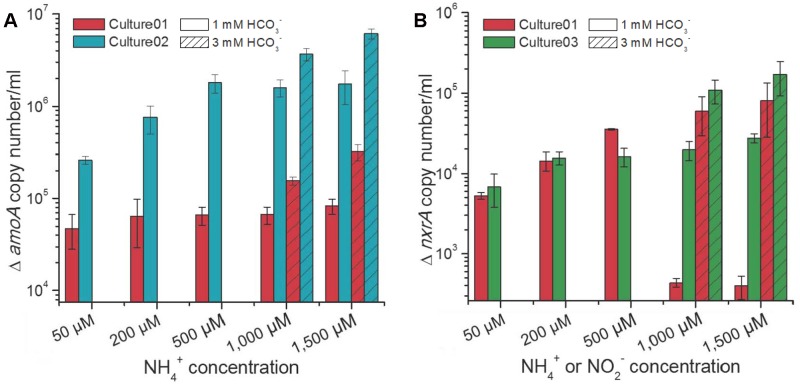
Increases in the **(A)** *amoA* and **(B)** *nxrA* gene copy numbers at different NH_4_^+^ or NO_2_^-^ concentrations. Culture01 and Culture02 were fed NH_4_^+^, while Culture03 was fed NO_2_^-^. The *amoA* gene copy numbers at time zero for Culture01 and Culture02 were 2.2 × 10^4^ ± 7.9 × 10^2^ and 4.0 × 10^4^ ± 1.3 × 10^3^ per ml, respectively. The *nxrA* gene copy number at time zero for Culture01 and Culture03 was 2.0 × 10^3^ ± 2.5 × 10^2^ per ml. Error bars represent one standard deviation from biological triplicate experiments.

The influences of different nitrogen and carbon concentrations on the growth of nitrifiers in the cultures were assessed by qPCR of the *amoA* and *nxrA* genes, representing the ammonia and nitrite oxidizers, respectively. Culture01 showed no increase in the extent of growth for the *Nitrosomonas*-like AOB with increasing NH_4_^+^ concentrations and no substantial growth for the *Nitrobacter*-like NOB under NH_4_^+^ concentrations of 1,000 and 1,500 μM when 1,000 μM HCO_3_^-^ was supplied (**Figure 4**). A HCO_3_^-^ concentration of 1,000 μM increased the growth of AOB in Culture02 at NH_4_^+^ concentration ≤500 μM and NOB in Culture03 at NO_2_^-^ concentrations ≤200 μM, but this growth became steady when nitrogen concentration increased (**Figure 4**). By contrast, an excess of HCO_3_^-^ (3,000 μM) promoted higher growth of AOB and NOB in all cultures (Student’s *t*-test, *p* < 0.01) when compared to cultures with 1,000 μM HCO_3_^-^ (**Figure 4**).

Under the same substrate (HCO_3_^-^ and NH_4_^+^) concentrations, the growth of AOB was significantly higher (Student’s *t*-test, *p* < 0.01) in Culture02 than in Culture01, with the *amoA* gene showing increases from 4.8 × 10^4^ ± 1.9 × 10^4^ to 3.2 × 10^5^ ± 6.3 × 10^4^ per ml for Culture01 and increases from 2.6 × 10^5^ ± 2.5 × 10^4^ to 6.1 × 10^6^ ± 7.5 × 10^5^ per ml for Culture02 (**Figure 4A**). Growth of the *Nitrobacter*-like NOB was similar between Culture01 and Culture03 when the HCO_3_^-^ was in excess, with increases in the *nxrA* gene ranging from 5.3 × 10^3^ ± 5.0 × 10^2^ to 8.0 × 10^4^ ± 3.8 × 10^4^ per ml culture for Culture01 and from 6.8 × 10^3^ ± 3.0 × 10^3^ to 1.6 × 10^5^ ± 7.7 × 10^4^ per ml for Culture03 (**Figure 4B**).

## Discussion

In this study, a biofilter from an aquarium was used to culture AOB and NOB, which have functional roles in removing NH_3_ and NO_2_^-^ that are toxic to aquatic life (Keuter et al., 2011; French et al., 2012; Wu et al., 2013). A culture-dependent approach was adopted to allow testing of the bacterial physiology. In particular, nitrification activities were examined with and without the synergistic interactions between the AOB and NOB. A 15-month cultivation period (over 60 transfers) with NH_4_^+^ as the sole energy source for the nitrifying community successfully cultured a number of AOB and NOB, as confirmed by both 16S rRNA gene amplicon and metagenomic sequencing. Culture02, cultured using chlorate, contained a few *Nitrobacter* 16S rRNA gene sequences (Supplementary Figure S1), but the NOB were unlikely to be functional as stoichiometric conversion of NH_4_^+^ to NO_2_^-^ was obtained and the *nxrA* gene was not amplified via PCR (Supplementary Table S1). The AOA belonging to *Nitrososphaera* were initially found in the biofilter, but no archaeal *amoA* gene or 16S rRNA gene was subsequently detected in the cultures. The relatively low culturing temperature (25°C as opposed to the 37°C preferred by AOA) (Wu et al., 2013), short retention time (Xia et al., 2011), high NH_4_^+^ concentration (Martens-Habbena et al., 2009; Ke et al., 2013) and high dissolved oxygen levels (Yan et al., 2012) likely favored AOB over AOA. We attempted to use ampicillin to isolate AOA with 500 μM NH_4_^+^ in synthetic medium (Mosier and Francis, 2008), but we detected no nitrification activity after incubating for more than one month.

Similar to the biofilter, the other major bacteria present in the cultures were from the *Acidovorax* lineage (Supplementary Figures S1, S7), which could reduce NO_3_^-^ under aerobic conditions at 20°C in the presence of polycyclic aromatic hydrocarbons (Eriksson et al., 2003), and different classes of *Proteobacteria* (Supplementary Figure S1). Despite the high oxygen concentration in the medium (>5.6 mg/L), anaerobic bacteria from the *Desulfitobacterium* lineage (Supplementary Figures S1, S7) remained in the cultures, similar to the biofilter [some *Desulfitobacterium* strains can exhibit high tolerance toward oxygen stress (De Wildeman et al., 2004; Kim et al., 2012)]. No nitrification-related genes were found in populations other than the AOB and NOB.

### Physiology of the Cultured AOB

The AOB in the cultures were assigned to the *Nitrosomonas* lineage (**Figure 2**), corroborating previous studies confirming that the bacteria in this lineage can typically function in environments with a low NH_4_^+^ concentration, such as soils (Wang et al., 2015), lake sediments (French et al., 2012), biofilms (Stehr et al., 1995), and freshwater aquarium biofilters (Burrell et al., 2001). The OTU with the most reads assigned to the AOB in the cultures (OTU01) showed the closest match (98% sequence identity) to the 16S rRNA gene of *N.* *oligotropha* Nm45, which grows at a relatively low NH_4_^+^ concentrations (1,000–5,000 μM) (Prosser et al., 2014) and has one of the highest affinities for NH_4_^+^ of all AOB studied (Koops et al., 1991; Martens-Habbena et al., 2009; Kits et al., 2017). Similarly, the hallmark genes (*amoCAB* and *haoA*) and the gene loci in the draft genomes of the AOB show high similarity to the two known species (i.e., *N.* *ureae* Nm10 and *Nitrosomonas* sp. AL212, Supplementary Figure S3) in the *N.* *oligotropha* lineage normally found in low NH_4_^+^ habitats, such as freshwater rivers and lakes (Koops et al., 1991; Prosser et al., 2014).

The growth rates (0.009–0.028 h^-1^) of the *Nitrosomonas*-like AOB in the cultures are within the range of those reported for other AOB (0.002–0.088 h^-1^) (French et al., 2012; Prosser and Nicol, 2012) and are most similar to the strains within *N. oligotropha* (0.02–0.06 h^-1^); this included the growth rate characteristic of no further increases at the NH_4_^+^ concentration of ∼500 μM (French et al., 2012). Growth rates of nitrifiers are closely related to inorganic carbon concentration as it has been shown that the change in HCO_3_^-^ concentrations can lead to greater changes in growth rate than NH_3_ and oxygen (Wett and Rauch, 2003; Jiang et al., 2015), indicating that the availability of inorganic carbons is a major factor that influences nitrification in freshwater environments. The higher growth rates observed at the NH_4_^+^ concentrations of 1,000 and 1,500 μM with 3 mM HCO_3_^-^ are likely the results of availability of additional inorganic carbons to the nitrifiers and a stronger capacity to buffer the protons released during NH_3_ oxidation (Ahn, 2006).

Previous studies have shown that the *K*_s_ of NH_4_^+^ for species within the *Nitrosomonas* lineage ranges from 30–3,900 μM, with strains of *N. oligotropha* having the lowest values (30–118 μM) among the reported AOB (Suwa et al., 1994; Martens-Habbena et al., 2009). Although the present case was not a pure culture, the *K*_s_ of NH_4_^+^ for Culture01 (25.9 μM) is lower than the previously reported values and the *K*_s_ for Culture02 is within the range reported for *N.* *oligotropha* (Supplementary Figure S8), suggesting that the AOB in the cultures are competitive in an oligotrophic environment. However, the affinity for NH_4_^+^ is definitely far weaker than the recently reported comammox bacterium *Nitrospira inopinata* (0.84 μM) (Kits et al., 2017).

Similar to the closely related *Nitrosomonas* sp. AL212 and *N.* *ureae* Nm10 (Yuichi et al., 2011; Kozlowski et al., 2016), the cultured *Nitrosomonas*-like AOB may not be obligately lithoautotrophic, as inference from the genome suggests they may be able to potentially use both organic and inorganic carbon (Supplementary Figure S5), which has to be further verified experimentally.

### Physiology of the Cultured NOB

For the nitrite oxidizers, five OTUs related to *Nitrobacter* and three OTUs to *Nitrospira* were detected in the biofilter. After cultivation, one of the more dominant nitrite oxidizers was OTU05, which shared 100% sequence identity to the 16S rRNA gene of *Candidatus* Nitrospira Defluvii [found in activated sludge (Spieck et al., 2006) and can survive under low NO_2_^-^ conditions (Lücker et al., 2010)]. *Nitrospira* are speculated to represent the most prevalent nitrite oxidizers in low-nitrogen environments, such as soils (Xia et al., 2011; Wang et al., 2015) and freshwater systems (Hovanec et al., 1998; Regan et al., 2002). We were unable to extract the *Nitrospira* genome in the cultures from the metagenomes, possibly due to its low abundance [Supplementary Table S8; confirmed by weak or no band in PCR (Supplementary Table S1)]. The other dominant NOB are related to OTU09, which clusters closely (100% 16S rRNA gene sequence identity) to the species *N.* *vulgaris* Z, one of the slowest lithoautotrophically growing nitrite oxidizers (doubling time 140 h) widely present in soils and freshwater (Bock et al., 1990). The *nxrAB* genes of the *Nitrobacter*-like NOB in the cultures are also highly similar to those found in *N.* *vulgaris* (Supplementary Figure S4); however, the genome of *N.* *vulgaris* is not available for whole genome-based phylogenetic analysis. The draft genome of Culture01_Bin8 represents the first sequenced genome of the species *N.* *vulgaris*. The nitrite reductase gene (*nirK*) was present in the draft genomes of the *Nitrosomonas*-like AOB and *Nitrobacter*-like NOB and confirmed via PCR (Supplementary Table S1); however, this gene is unlikely to be functional in our study, since we found a stoichiometric conversion of NH_4_^+^ to NO_2_^-^ in our cultures (**Figure 1**). In addition, our cultivation was performed in an oxygen-rich condition (>5.6 mg/L), contrary to previous studies that showed functioning of the *nirK* gene in a low oxygen environment (0–4%) (Starkenburg et al., 2008; Attard et al., 2010).

*Nitrobacter* NOB are usually of high abundance in environments with a high nitrogen content, such as wastewater (Wagner and Loy, 2002) and high-nutrient soils (Attard et al., 2010). The accumulated NO_2_^-^ concentration (maximum 355 μM) in Culture01 is not considered high; nevertheless, the *Nitrobacter*-like NOB was much more abundant than *Nitrospira* (**Figure 2** and Supplementary Figure S1). This contrasts with other aquarium biofilter studies that found *Nitrospira*-like NOB to be the most common nitrite oxidizers (Hovanec et al., 1998; Burrell et al., 2001). This difference could be due to the likelihood of a faster growth rate of the *Nitrobacter*-like NOB in our cultures, as *N. vulgaris* has a short generation time (13 h) compared with other species of NOB (∼26–43 h) (Nowka et al., 2015) and the *K*_s_ of NO_2_^-^ is lower for *N. vulgaris* (49 μM) than for other *Nitrobacter*-like NOB and is similar to *Nitrospira* (Nowka et al., 2015), suggesting that *N. vulgaris* can be as competitive as *Nitrospira* in oligotrophic environments.

### Synergistic Interactions between the AOB and NOB in the Cultures

AOB and NOB coexist in both natural (e.g., rice paddy soils) (Ke et al., 2013; Wang et al., 2015) and engineered systems (e.g., wastewater treatment plants) (Dionisi et al., 2002), but previous studies investigating their relationships were usually carried out using artificially constructed co-cultures of AOB and NOB isolates (Laanbroek and Gerards, 1993; Perez et al., 2015). These approaches may not reflect the nitrification processes in a real-life ecosystem, and most studies also tended to focus on nitrifiers in activated sludge (Sliekers et al., 2005; Winkler et al., 2012), with fewer co-culture studies on members of freshwater biofilters (van Kessel et al., 2010).

Metagenomic and 16S rRNA gene sequencing, together with physiological experiments, indicated that the nitrifying partnership in the cultures involves a *Nitrosomonas*-like AOB and a *Nitrobacter*-like NOB that are adapted to a relatively low NH_4_^+^ condition. The combination of AOB and NOB in Culture01 reduced the lag time compared to that in Culture02 and Culture03. For example, incubation at the lowest NH_4_^+^ or NO_2_^-^ concentration tested (50 μM) gave longer lag phases for Culture02 and Culture03 than for Culture01 of 10- and 3.5-fold, respectively. Although nitrifiers are reportedly sensitive to the initial incubation conditions (e.g., substrate concentrations) (Graham et al., 2007), the duration of the lag phase also varies significantly when incubated with the same NH_4_^+^ concentration, depending on whether the nitrifying partner is present. Furthermore, similar to a previous study (Sedlacek et al., 2016), the growth rate also highly depends on the presence of the nitrifying partner, with Culture01 showing the highest rate compared to the two other cultures, and especially Culture03 (**Figure 3A**). The extent of cell growth for the AOB was lower in Culture01 than in Culture02 and the NOB in Culture 01 had similar growth to that seen in Culture03 (**Figure 4**). The major difference between Culture01 and Culture02 was the composition of the nitrifiers; therefore, the decoupling between the growth of AOB and nitrification activities might reflect how the cells utilize the energy derived from NH_4_^+^ in the presence NOB. In line with the previous finding that AOB had a higher *K*_s_ value for NH_4_^+^ in the absence of NOB (Sedlacek et al., 2016), the *K*_s_ of NH_4_^+^ for Culture02 without NOB was threefold higher than for Culture01 with NOB, suggesting that the synergistic interactions between AOB and NOB enable the robust oxidation of NH_4_^+^ at a low concentration. The presence of heterotrophic bacteria with AOB had also been shown to increase the expression of proteins related to the ammonia oxidation pathway of AOB and promote nitrification (Sedlacek et al., 2016). The kinetic parameters and the synergistic relationships can potentially be used to enhance nutrient removal in freshwater treatment systems, which could benefit aquatic life and prevent eutrophication (Hou et al., 2013).

The physical contact between AOB and NOB allows the efficient transfer of compounds between them (Okabe et al., 1999). Reciprocal feeding between AOB and NOB has been experimentally demonstrated by co-culturing AOB and NOB, whereby *Nitrospira* *moscoviensis* breaks down urea or cyanate to NH_4_^+^ to provide an energy source for *N.* *europaea* or *Nitrosomonas* *nitrosa*, which in turn produce NO_2_^-^ for the NOB (Palatinszky et al., 2015). This source of NH_4_^+^ is important for the functioning of AOB in oligotrophic environments (Prosser et al., 2014). Interestingly, the gene encoding cyanate hydratase (Starkenburg et al., 2006), which converts cyanate to NH_3_ and CO_2_ in the presence of HCO_3_^-^, is found in the genome of the *Nitrobacter*-like NOB (Culture01_Bin8) (also present in *N.* *winogradskyi* Nb-255), and this could aid in supporting AOB when urea or cyanate is available.

## Conclusion

We used a culture-dependent approach to simulate the *in situ* nitrification process in a freshwater biofilter to study the ecophysiology of the ammonia-oxidizing and nitrite-oxidizing guilds at a relatively low nitrogen concentration and the synergistic relationships between these guilds. The cultured *N.* *vulgaris* may be as competitive as *Nitrospira*-like NOB in oligotrophic environments. The nitrification kinetics of the cultures are influenced by NH_4_^+^ and/or NO_2_^-^, and HCO_3_^-^ concentrations. Metagenomic sequencing indicated the draft genomes of the nitrifying partners (*Nitrosomonas*-like AOB and *Nitrobacter*-like NOB), and that their growth rate, substrates affinity, and lag duration strongly depended on the presence of each partner. Although the AOB and NOB could function independently, when both were present together, robust nitrification occurred. Overall, the observations in this study indicate the competitiveness of the cultured *Nitrosomonas*-like AOB and *Nitrobacter*-like NOB in an oligotrophic environment and a strong dependence of their activities on the synergistic relationships between the two guilds. These results provide insights for possible manipulations of multi-species interactions to optimize nitrification treatment processes.

## Author Contributions

MC performed the research, analyzed the data, and wrote the manuscript. S-KN, CKL, HL, and YJ performed the research and analyzed the data. PKHL designed the experiments and wrote the manuscript.

## Conflict of Interest Statement

The authors declare that the research was conducted in the absence of any commercial or financial relationships that could be construed as a potential conflict of interest.
