# Supplementary material for: Physiological and Metagenomic Characterizations of the Synergistic Relationships between Ammonia- and Nitrite-Oxidizing Bacteria in Freshwater Nitrification
Source: Front Microbiol. 2018 Feb 27;9:280. doi: 10.3389/fmicb.2018.00280 (PMC5835065; doi:10.3389/fmicb.2018.00280)
Supplement: Supplementary file 1 [file Data_Sheet_1.DOC]

**Supporting Information**

**Physiological and metagenomic characterizations of the synergistic relationships between ammonia- and nitrite-oxidizing bacteria in freshwater nitrification**

Mingwei Cai, Siu-Kin Ng, Chee Kent Lim, Hongyuan Lu, Yangyang Jia, and Patrick K. H. Lee*

School of Energy and Environment, City University of Hong Kong, Tat Chee Avenue, Kowloon, Hong Kong

**List of tables**

Table S1. Primers used for qPCR and PCR amplifications

Table S2. Similarity between the bins recovered in Culture01 and Culture02

Table S3. Summary of *de* *novo* assembly of the six metagenomes

Table S4. Characteristics of the genomic bins

Table S5. Similarity between the genome bins obtained using MaxBin and differential coverage binning

Table S6. Putative functions of ORFs of Bin1 in Culture01

Table S7. Putative functions of ORFs of Bin8 in Culture01

Table S8. Relative composition of the nitrifiers in Culture01 and Culture02 according to different methods

**List of figures**

Fig. S1. Relative abundance of the top 10 genera detected in the biofilter, Culture01, and Culture02 samples

Fig. S2. Binning of the draft genomes in (A) Culture01 [(B) zoomed region in (A)] and (C) Culture02 [(D) zoomed region in (C)] using the differential coverage approach based on multidimensional scaling (MDS) of the three metagenomes

Fig. S3. Key nitrification gene loci of the cultured nitrifiers and other neighboring species as well as the known comammox *Nitrospira* bacteria

Fig. S4. Phylogenetic position of the hallmark genes for the two-step nitrification

Fig. S5. Reconstruction of the key carbon metabolic pathways in the genomes of the cultured nitrifiers and the closest references (selected according to PhyloPhlAn, Fig. 2)

Fig. S6. Gene function map comparing the contigs assigned to genomic (A) Bin1 and (B) Bin8 (left side) against the genome of (A) *Nitrosomonas* sp. AL212 and (B) *Nitrobacter winogradskyi*Nb-255 (right side)

Fig. S7. Phylogenetic position of the OTU belonging to (A) *Desulfitobacterium* and (B) *Acidovorax* in Culture01 and Culture02

Fig. S8. K_s_ values comparison between different reported AOB isolates or enrichments

**Table S1.** Primers used for qPCR and PCR amplifications.

| **Target gene ^a^** | **Target species** | **Primer** | **Sequence (5’ to 3’)** | **PCR product size (bp)** | **Reference** | **Expected band ^b^** |
| --- | --- | --- | --- | --- | --- | --- |
| *amoA*  (NH_4_^+^ to NH_2_OH) | AOA | Arch-amoAF | STAATGGTCTGGCTTAGACG | 635 |  | Culture01 (no band) Culture02 (no band) |
|  |  | Arch-amoAR | GCGGCCATCCATCTGTATGT |  |  |  |
|  |  |  |  |  |  |  |
|  | AOB | amoA-1F | GGGGTTTCTACTGGTGGT | 491 |  | Culture01 (yes) Culture02 (yes) |
|  |  | amoA-2R | CCCCTCKGSAAAGCCTTCTTC |  |  |  |
|  |  |  |  |  |  |  |
| 16S rRNA gene | *Nitrospira*-like NOB | Nspra-675f | GCGGTGAAATGCGTAGAKATCG | 71 |  | Culture01 (weak band) Culture02 (no band) |
|  |  | Nspra-746r | TCAGCGTCAGRWAYGTTCCAGAG |  |  |  |
|  |  |  |  |  |  |  |
| *nxrA*  (NO_2_^-^ to NO_3_^-^) | *Nitrobacter*-like NOB | F1norA | CAGACCGACGTGTGCGAAAG | 322 |  | Culture01 (yes) Culture02 (no band) |
|  |  | R2norA | TCCACAAGGAACGGAAGGTC |  |  |  |
|  |  |  |  |  |  |  |
| *nirK*  (NO_2_^-^ to NO) | Nitrite  oxidizers | nirK876F | ATYGGCGGVCAYGGCGA | 165 |  | Culture01 (weak band) Culture02 (weak band) |
|  |  | nirK1040R | GCCTCGATCAGRTTRTGGTT |  |  |  |

^a^ The average qPCR efficiency was 98.3% for the bacterial *amoA* gene and 91.6% for the *nxrA* gene (R^2^ value ≥ 0.99)

^b^ PCR results

**Table S2.** Similarity between the bins recovered in Culture01 and Culture02.

| **Culture01 ^a^** | **Culture02 ^a^** | **Genus** | **Genome size of bins in Culture01**  **(Mb) ^b^** | **Genome size of bins in Culture02**  **(Mb) ^b^** | **Total Alignment length**  **(Mb) ^c^** | **Similarity**  **(Against Culture01 as reference)** **^d^**  **(%)** | **Similarity**  **(Against Culture02 as reference) ^d^**  **(%)** |
| --- | --- | --- | --- | --- | --- | --- | --- |
| Bin1 | Bin3 | *Nitrosomonas* | 3.0 | 3.0 | 3.0 | 98.0 | 99.1 |
| Bin2 | Bin5 | *Bosea* | 5.7 | 5.7 | 5.0 | 88.1 | 88.1 |
| Bin3 | Bin1 | *Sediminibacterium* | 3.7 | 3.7 | 3.7 | 99.3 | 99.8 |
| Bin4 | Bin6 | *Hydrogenophaga* | 5.6 | 5.6 | 5.5 | 99.3 | 99.5 |
| Bin5 | Bin2 | *Pseudomonas* | 6.3 | 6.3 | 4.9 | 77.3 | 76.5 |

^a^ Genome bins were obtained using differential coverage binning

^b^ Genome size was estimated by the total length of contigs

^c^ Pairwise comparison of genome bins was done using BLASTn. Unique BLAST hits with alignment length ≥ 5 kb were used to calculate the total alignment length

^d^ Genome-wise similarity was obtained from the equation: (total alignment length × mean identity of alignments) / genome size. As sequence identities of almost all BLASTn hits were close to 100%, 100% was used as the mean identity of alignments when calculating genome-wise similarity

**Table S3.** Summary of *de* *novo* assembly of the six metagenomes.^a^

| **Sample** | **Metagenome size**  **(Gb)** | **Average read length**  **(bp)** | **Total size of the contigs**  **(Mb)** | **Average contig length**  **(kb)** | **Longest contig length**  **(kb)** | **N50**  **(kb)** |
| --- | --- | --- | --- | --- | --- | --- |
| Culture01_1 | 2.8 | 126 | 45.7 | 22.7 | 271.6 | 92.0 |
| Culture01_3 | 1.9 | 126 | 48.0 | 9.4 | 593.5 | 37.8 |
| Culture01_5 | 1.9 | 125 | 52.2 | 9.3 | 1,065.8 | 31.1 |
| Culture02_1 | 2.6 | 126 | 50.2 | 11.1 | 770.1 | 97.0 |
| Culture02_3 | 2.2 | 125 | 54.4 | 12.5 | 638.0 | 81.1 |
| Culture02_5 | 1.0 | 126 | 59.2 | 7.6 | 457.3 | 62.3 |

^a^ All values reported here refer to reads that passed quality control. Only contigs ≥ 1.2 kb were considered

**Table S4.** Characteristics of the genomic bins.

| **Bin** | **Marker lineage (genus)** | **Number of contigs** | **Genome size**  **(Mb)** | **N50**  **(kb)** | **Longest contig**  **(kb)** | **Completeness** **^a^**  **(%)** | **Contamination ^a^**  **(%)** | **Number of tRNA ^b^** |
| --- | --- | --- | --- | --- | --- | --- | --- | --- |
| Culture01_Bin1 | *Nitrosomonas* | 90 | 3.0 | 70.4 | 117.5 | 97.9 | 0 | 36 |
| Culture01_Bin2 | *Bosea* | 137 | 5.7 | 70.7 | 165.8 | 98.3 | 0.9 | 45 |
| Culture01_Bin3 | *Sediminibacterium* | 35 | 3.7 | 176.4 | 474.1 | 94.5 | 0 | 37 |
| Culture01_Bin4 | *Hydrogenophaga* | 65 | 5.6 | 154.3 | 417.8 | 99.2 | 0.8 | 41 |
| Culture01_Bin5 | *Pseudomonas* | 370 | 6.3 | 26.1 | 109.0 | 98.3 | 0.4 | 48 |
| Culture01_Bin6 | *Variovorax* | 354 | 9.2 | 15.1 | 116.3 | 94.1 | 2.4 | 47 |
| Culture01_Bin7 | *Acidovorax* | 30 | 4.2 | 190.9 | 593.4 | 99.1 | 1.7 | 42 |
| Culture01_Bin8 | *Nitrobacter* | 159 | 3.9 | 40.2 | 140.8 | 100 | 1.7 | 50 |
|  |  |  |  |  |  |  |  |  |
| Culture02_Bin1 | *Sediminibacterium* | 50 | 3.7 | 104.9 | 395.8 | 94.5 | 0 | 36 |
| Culture02_Bin2 | *Pseudomonas* | 119 | 6.3 | 85.0 | 326.9 | 98.3 | 0 | 50 |
| Culture02_Bin3 | *Nitrosomonas* | 81 | 3.0 | 70.4 | 117.5 | 97.0 | 0 | 38 |
| Culture02_Bin4 | *Shinella* | 75 | 6.7 | 121.3 | 407.2 | 100 | 0 | 48 |
| Culture02_Bin5 | *Bosea* | 64 | 5.7 | 175.2 | 638.0 | 98.3 | 0.9 | 46 |
| Culture02_Bin6 | *Hydrogenophaga* | 53 | 5.6 | 186.5 | 390.6 | 99.2 | 0 | 41 |
| Culture02_Bin7 | *Bradyrhizobium* | 267 | 5.3 | 25.4 | 94.2 | 87.8 | 1.4 | 43 |
| Culture02_Bin8 | *Cupriavidus* | 45 | 6.1 | 188.9 | 373.6 | 100 | 0.8 | 50 |
| Culture02_Bin9 | *Mesorhizobium* | 486 | 4.5 | 9.7 | 40.4 | 69.5 | 0 | 52 |

^a^ Completeness and contamination were evaluated using CheckM (version 1.0.4)

^b^ Prediction of tRNA sequences were performed with tRNAscan-SE (version 1.4)

**Table S5.** Similarity between the genome bins obtained using MaxBin and differential coverage binning.

| **Culture01** | **Genus** | **Differential coverage binning** | | |  | **MaxBin** | | |  | **Shared contigs** | |
| --- | --- | --- | --- | --- | --- | --- | --- | --- | --- | --- | --- |
|  |  | **Number of Contig ^a^** | **Genome size**  **(Mb) ^b^** | **Coverage**  **(%) ^c^** |  | **Number of Contig ^a^** | **Genome size**  **(Mb) ^b^** | **Coverage**  **(%) ^c^** |  | **Number of Shared contigs ^d^** | **Total length**  **(Mb) ^e^** |
| Bin1 | *Nitrosomonas* | 90 | 3.0 | 98.4 |  | 76 | 3.0 | 100 |  | 76 | 3.0 |
| Bin2 | *Bosea* | 137 | 5.7 | 89.8 |  | 122 | 5.3 | 97.6 |  | 114 | 5.1 |
| Bin3 | *Sediminibacterium* | 35 | 3.7 | 99.7 |  | 33 | 3.7 | 99.7 |  | 32 | 3.7 |
| Bin4 | *Hydrogenophaga* | 65 | 5.6 | 97.0 |  | 53 | 5.4 | 99.1 |  | 51 | 5.4 |
| Bin5 | *Pseudomonas* | 370 | 6.3 | 90.7 |  | 339 | 6.7 | 85.0 |  | 289 | 5.7 |
| Bin6 | *Variovorax* | 854 | 9.2 | 73.9 |  | 513 | 6.8 | 99.8 |  | 511 | 6.1 |
| Bin7 | *Acidovorax* | 30 | 4.2 | 99.8 |  | 42 | 4.4 | 94.6 |  | 30 | 4.2 |
| Bin8 | *Nitrobacter* | 159 | 3.9 | 94.9 |  | 199 | 5.0 | 72.7 |  | 131 | 3.7 |

^a^ The number of contigs (from sequence template) assigned to a genome bin using different binning methods (differential coverage binning or MaxBin)

^b^ Genome size was estimated by the total length of contigs

^c^ Coverage (%) was obtained from: total length of shared contigs/genome size

^d^ Shared contigs were the contigs simultaneously assigned to the same bin using the two binning methods

^e^ Total length of shared contigs

**Table S8.** Relative composition of the nitrifiers in Culture01 and Culture02 according to different methods.^a^

| **Sample** | **Genus** | **16S rRNA gene ^b^**  **(%)** | **Contigs ^c^**  **(%)** | **Merged reads ^c^**  **(%)** | **ORFs ^d^**  **(%)** |  |
| --- | --- | --- | --- | --- | --- | --- |
| Culture01 | *Nitrosomonas* | 29.0 | 34.8 | 44.9 | 55.0 |  |
|  | *Nitrobacter* | 64.1 | 60.2 | 48.5 | 44.9 |  |
|  | *Nitrospira* | 6.9 | 3.6 | 5.1 | 0.1 |  |
|  | *Nitrosospira* | 0 | 0.9 | 1.1 | 0 |  |
|  | *Nitrosococcus* | 0 | 0.6 | 0.3 | 0 |  |
|  | *Nitrococcus* | 0 | 0 | 0.1 | 0 |  |
|  |  |  |  |  |  |  |
| Culture02 | *Nitrosomonas* | 89.6 | 74.8 | 91.5 | 89.2 |  |
|  | *Nitrobacter* | 10.0 | 21.7 | 5.3 | 10.8 |  |
|  | *Nitrospira* | 0.4 | 2.6 | 0.1 | 0 |  |
|  | *Nitrosospira* | 0 | 0.1 | 2.4 | 0 |  |
|  | *Nitrosococcus* | 0 | 0.7 | 0.4 | 0 |  |
|  | *Nitrococcus* | 0 | 0 | 0.2 | 0 |  |

^a^ Only the nitrifiers were considered in the relative abundance calculation

^b^ Based on amplicon sequencing

^c^ The annotation was performed in MG-RAST

^d^ Annotated against the NCBI non-redundant database

**
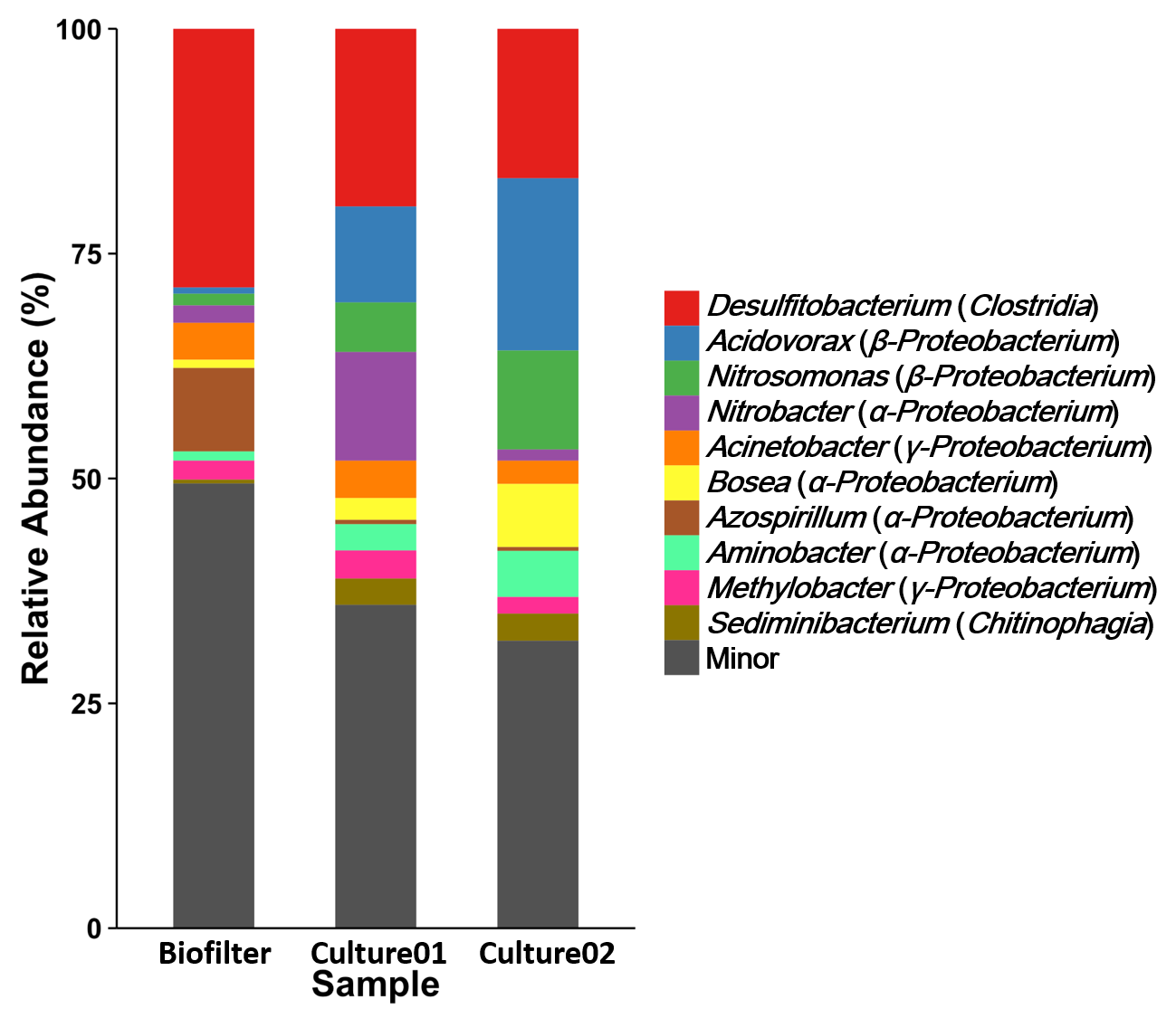
**

**Fig. S1.** Relative abundance of the top 10 genera detected in the biofilter, Culture01, and Culture02 samples. The corresponding class of the genera is shown in the brackets. The proportions were calculated based on the 16S rRNA gene data.


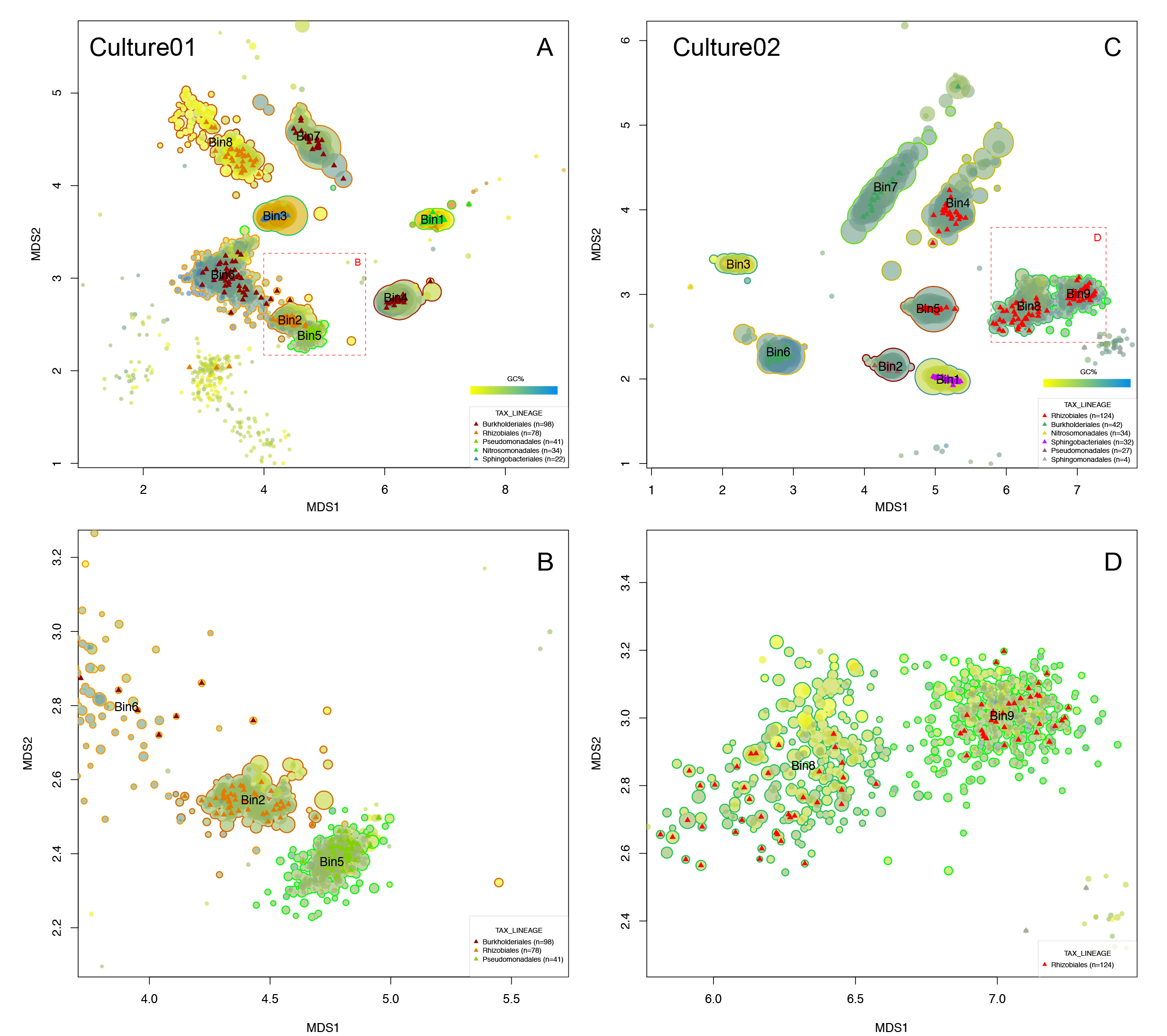


**Fig. S2.** Binning of the draft genomes in (A) Culture01 [(B) zoomed region in (A)] and (C) Culture02 [(D) zoomed region in (C)] using the differential coverage approach based on multidimensional scaling (MDS) of the three metagenomes. Circles represent contigs, scaled by the square root of their length and color-filled according to their GC%. Contigs belonging to the same genome bin are outlined with the same color. Color-coded triangles show taxonomic orders of the conserved marker proteins. Only contigs ≥ 1.2 kb are shown.

**
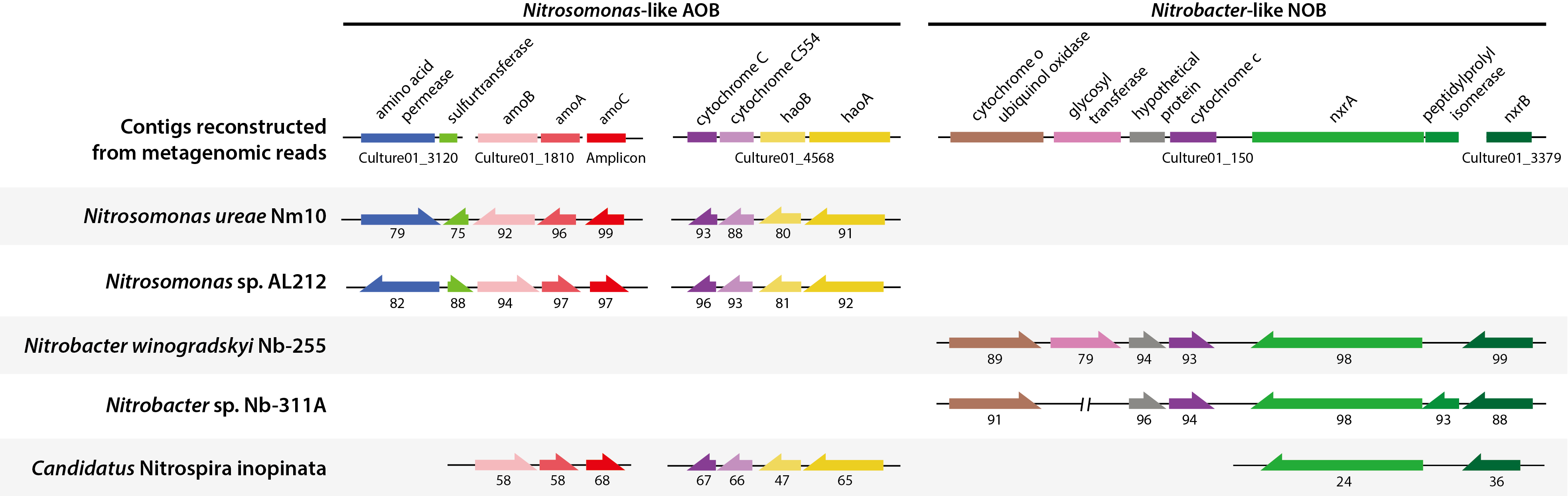
**

**Fig. S3.** Key nitrification gene loci of the cultured nitrifiers and other neighboring species as well as the known comammox *Nitrospira* bacteria. Alignments of the *amo*, *hao*, and *nxr* loci with flanking genes are shown. Colors identify homologous genes. The value below each gene represents amino acid similarity aligned against the reconstructed contigs using protein BLAST. Genes are drawn to scale.


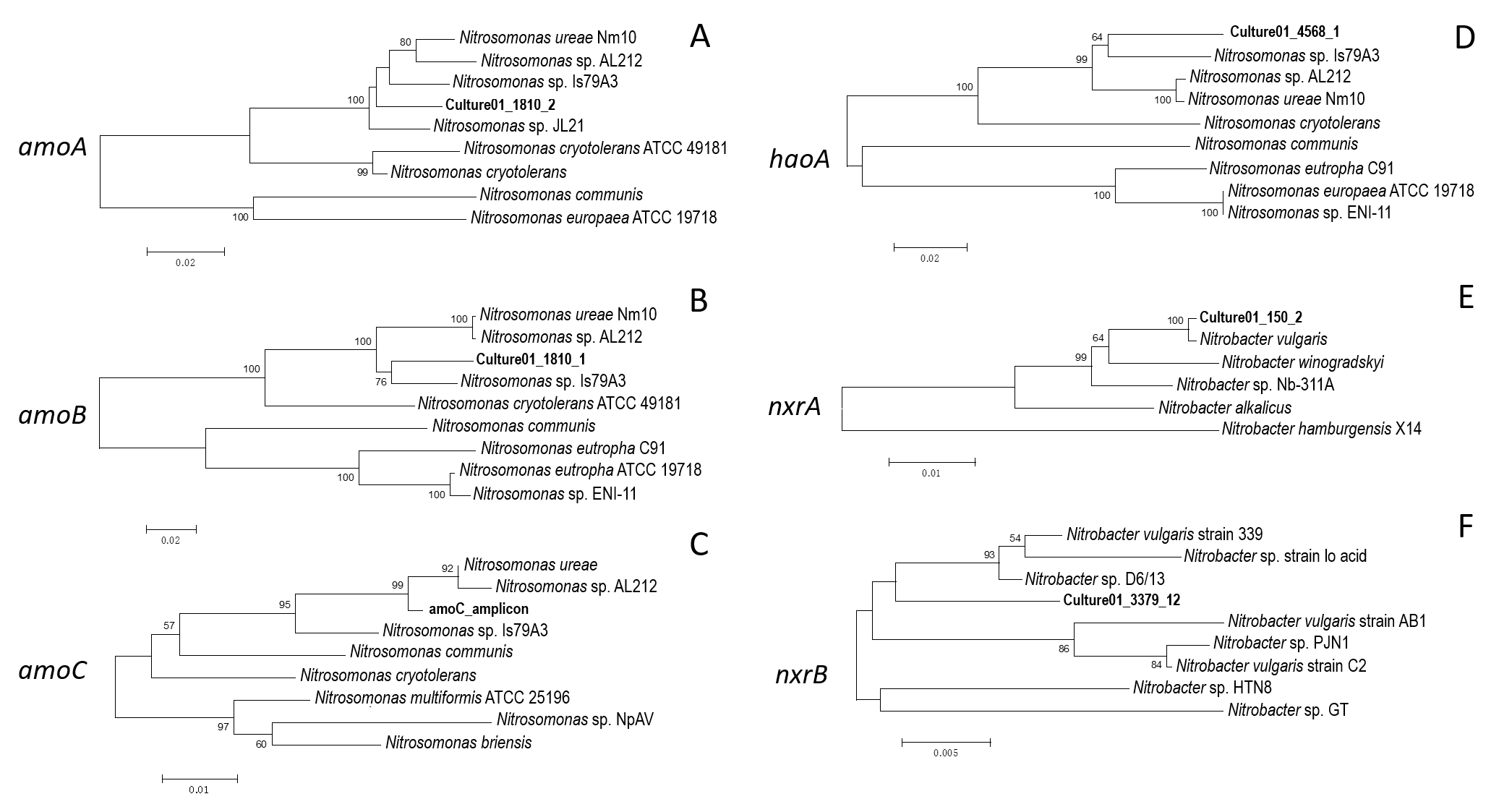


**Fig. S4.** Phylogenetic position of the hallmark genes for the two-step nitrification. The trees were built based on genetic distance of the protein sequences using the neighbor-joining algorithm of MEGA 5.2. Only bootstrap values (1,000 iterations) greater than 50% are shown.


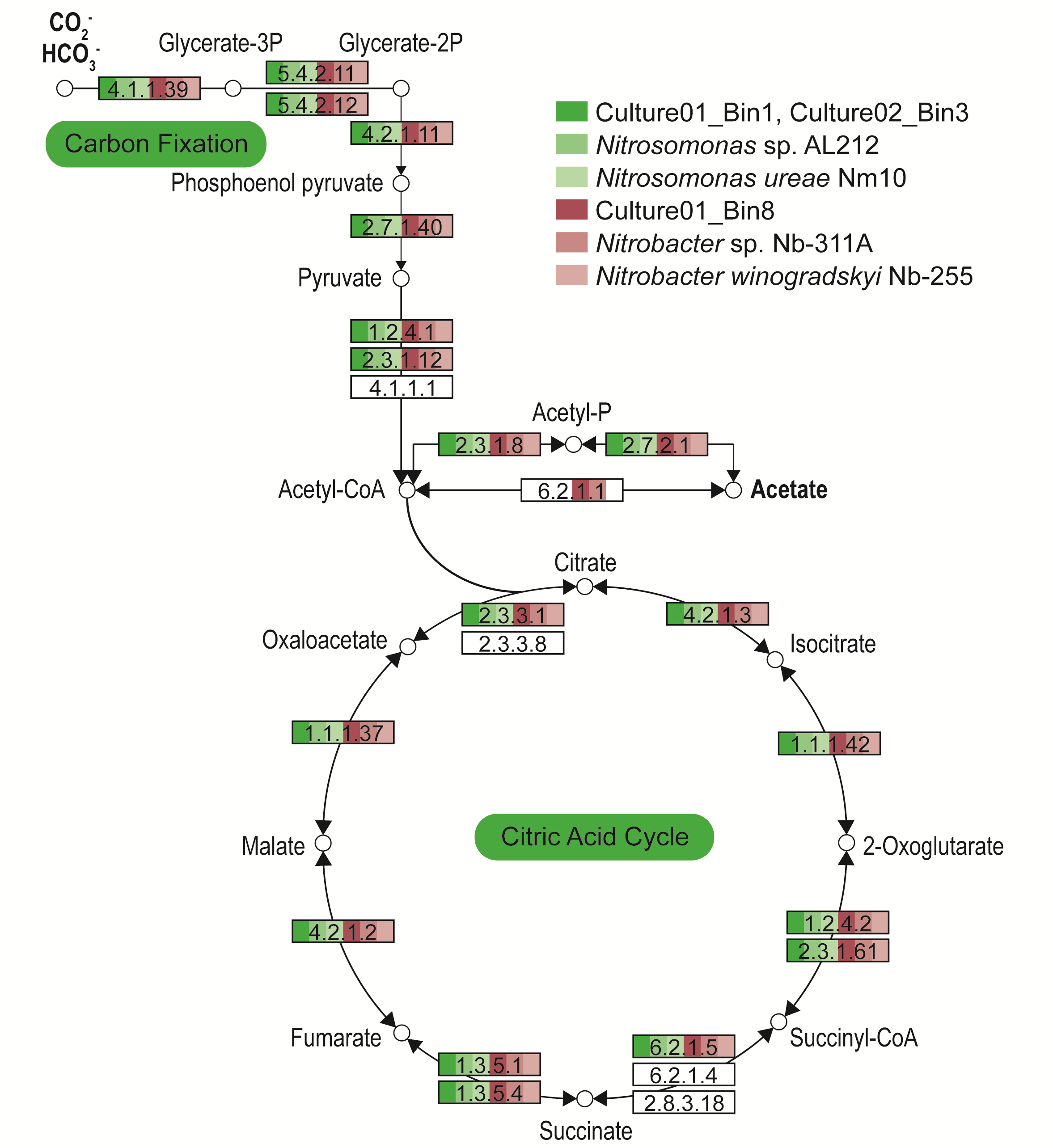


**Fig. S5.** Reconstruction of the key carbon metabolic pathways in the genomes of the cultured nitrifiers and the closest references (selected according to PhyloPhlAn, Fig. 2). The numbered rectangles represent different enzymes. The cultured nitrifiers were annotated against the KEGG database, while the reference genomes were based on both the KEGG and BRENDA databases.


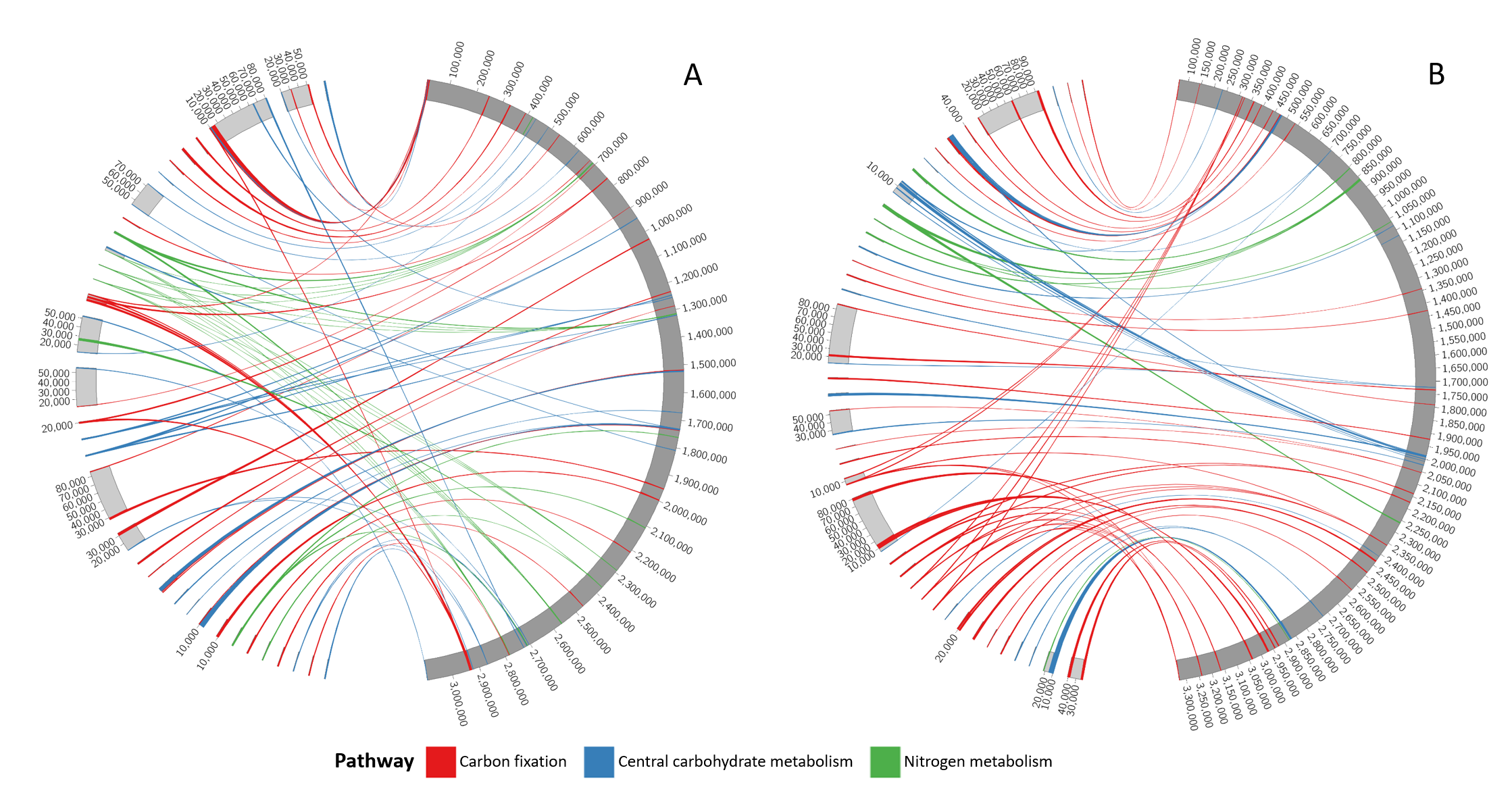


**Fig. S6.** Gene function map comparing the contigs assigned to genomic (A) Bin1 and (B) Bin8 (left side) against the genome of (A) *Nitrosomonas* sp. AL212 and (B) *Nitrobacter winogradskyi*Nb-255 (right side). The maps were drawn using Circos . ORFs annotated with KEGG orthology (KO) genes belonging to the carbon fixation (red), central carbohydrate metabolism (blue), or nitrogen metabolism (green) pathways are highlighted in each contig and are joined by colored ribbons to loci with matching KOs in the reference genome. Only ORFs or loci annotated with a KO in a pathway of interest and that have at least one matched locus or ORF are shown. Only contigs with at least one ORF of interest are shown. Sequences have been truncated to show only the ORFs and loci of interest; tick marks indicate the absolute base location within the untruncated sequence.


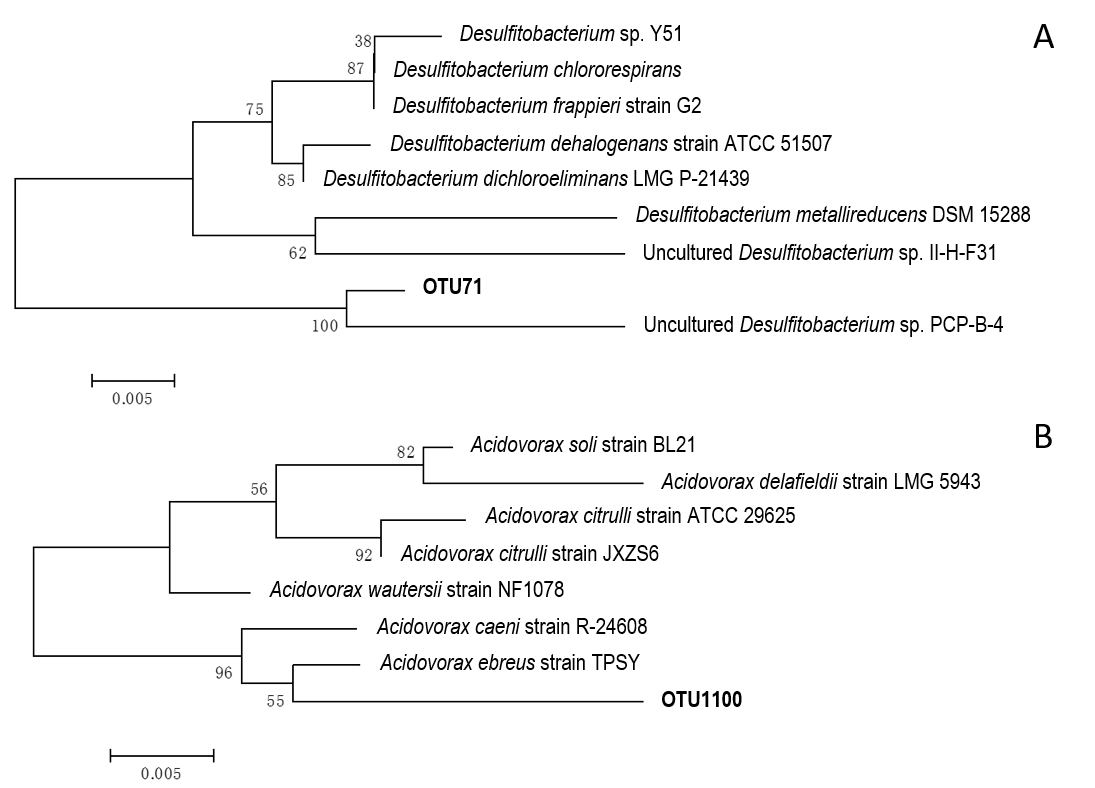


**Fig. S7.** Phylogenetic position of the OTU belonging to (A) *Desulfitobacterium* and (B) *Acidovorax* in Culture01 and Culture02. The trees were built based on the 16S rRNA gene sequences using the neighbor-joining algorithm of MEGA 5.2. Only bootstrap values (1,000 iterations) greater than 50% are shown.


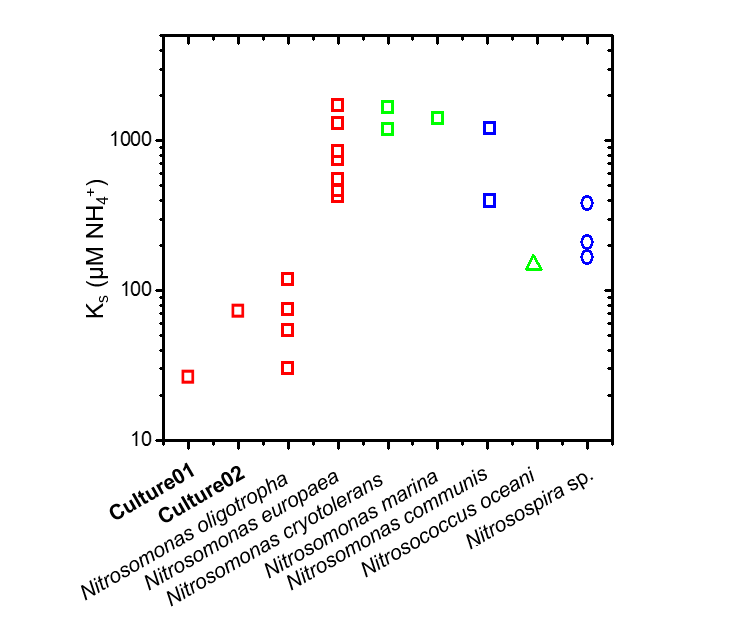


**Fig. S8.** K_s_ values comparison between different reported AOB isolates or enrichments . The environmental conditions under which the isolates or enrichments originated are indicated by color: freshwater (red), marine (green), and soil (blue). Symbols represent different genera.

**References**

Attard, E., Poly, F., Commeaux, C., Laurent, F., Terada, A., Smets, B. F., et al. (2010). Shifts between *Nitrospira*-and *Nitrobacter*-like nitrite oxidizers underlie the response of soil potential nitrite oxidation to changes in tillage practices. *Environmental Microbiology* 12, 315-326. doi: 10.1111/j.1462-2920.2009.02070.x

Braker, G., Fesefeldt, A., Witzel, K. P. (1998). Development of PCR primer systems for amplification of nitrite reductase genes (*nirK* and *nirS*) to detect denitrifying bacteria in environmental samples. *Appl. Environ. Microbiol.* 64, 3769-3775.

Francis, C. A., Roberts, K. J., Beman, J. M., Santoro, A. E., Oakley, B. B. (2005). Ubiquity and diversity of ammonia-oxidizing archaea in water columns and sediments of the ocean. *Proc. Natl. Acad. Sci. USA* 102, 14683-14688. doi: 10.1073/pnas.0506625102.

Graham, D. W., Knapp, C. W., Van Vleck, E. S., Bloor, K., Lane, T. B., Graham, C. E. (2007). Experimental demonstration of chaotic instability in biological nitrification. *ISME J.* 1, 385-393. doi: 10.1038/ismej.2007.45. doi: 10.1038/ismej.2007.45

Groeneweg, J., Sellner, B., Tappe, W. (1994). Ammonia oxidation in *Nitrosomonas* at NH_3_ concentrations near K_m_: effects of pH and temperature. *Water Res.* 28, 2561-2566. doi: 10.1016/0043-1354(94)90074-4

He, J., Hu, H., Zhang, L. (2012). Current insights into the autotrophic thaumarchaeal ammonia oxidation in acidic soils. *Soil Biol. Biochem.* 55, 146-154. doi: 10.1016/j.soilbio.2012.06.006

Jung, M., Park, S., Min, D., Kim, J., Rijpstra, W. I. C., Damsté, J. S. S., et al. (2011). Enrichment and characterization of an autotrophic ammonia-oxidizing archaeon of mesophilic crenarchaeal group I. 1a from an agricultural soil. *Appl. Environ. Microbiol.* 77, 8635-8647. doi: 10.1128/AEM.05787-11

Koops, H., Pommerening Röser, A. (2001). Distribution and ecophysiology of the nitrifying bacteria emphasizing cultured species. *FEMS Microbiol. Ecol.* 37, 1-9. doi: 10.1111/j.1574-6941.2001.tb00847.x

Krzywinski, M., Schein, J., Birol, I., Connors, J., Gascoyne, R., Horsman, D., et al. (2009). Circos: an information aesthetic for comparative genomics. *Genome Res.* 19, 1639-1645. doi: 10.1101/gr.092759.109

Park, B., Park, S., Yoon, D., Schouten, S., Damsté, J. S. S., Rhee, S. (2010). Cultivation of autotrophic ammonia-oxidizing archaea from marine sediments in coculture with sulfur-oxidizing bacteria. *Appl. Environ. Microbiol.* 76, 7575-7587. doi: 10.1128/AEM.01478-10

Pommerening-Röser, A., Rath, G., Koops, H.-P. (1996). Phylogenetic diversity within the genus *Nitrosomonas*. *Syst. Appl. Microbiol.* 19, 344-351. doi: 10.1016/S0723-2020(96)80061-0

Prosser, J. I., Head, I. M., Stein, L. Y. (2014). "The family *Nitrosomonadaceae*," in *The Analysis of Gene Expression Data*, eds E. Rosenberg, E. F. DeLong, S. Lory, E. Stackebrandt and F. Thompson (Springer Berlin Heidelberg), 901-918.

Rotthauwe, J. H., Witzel, K. P., Liesack, W. (1997). The ammonia monooxygenase structural gene amoA as a functional marker: molecular fine-scale analysis of natural ammonia-oxidizing populations. *Appl. Environ. Microbiol.* 63, 4704-4712.

Taylor, A. E., Bottomley, P. J. (2006). Nitrite production by *Nitrosomonas europaea* and *Nitrosospira* sp. AV in soils at different solution concentrations of ammonium. *Soil Biol. Biochem.* 38, 828-836. doi: 10.1016/j.soilbio.2005.08.001

Ward, B. (1987). Kinetic studies on ammonia and methane oxidation by *Nitrosococcus oceanus*. *Arch. Microbiol.* 147, 126-133.
